# Understanding Molecular Mechanisms of the Brain Through Transcriptomics

**DOI:** 10.3389/fphys.2019.00214

**Published:** 2019-03-15

**Authors:** Wei Wang, Guang-Zhong Wang

**Affiliations:** Key Laboratory of Computational Biology, CAS-MPG Partner Institute for Computational Biology, Shanghai Institute of Nutrition and Health, Shanghai Institutes for Biological Sciences, University of Chinese Academy of Sciences, Chinese Academy of Sciences, Shanghai, China

**Keywords:** brain transcriptome, WGCNA, neurodevelopmental disorders, differentially expressed genes, cerebral cortex

## Abstract

The brain is the most complicated organ in the human body with more than ten thousand genes expressed in each region. The molecular activity of the brain is divergent in various brain regions, both spatially and temporally. The function of each brain region lies in the fact that each region has different gene expression profiles, the possibility of differential RNA splicing, as well as various post-transcriptional and translational modification processes. Understanding the overall activity of the brain at the molecular level is essential for a comprehensive understanding of how the brain works. Fortunately, the development of next generation sequencing technology has made it possible to measure the molecular activity of a specific tissue as a daily routine approach of research. Therefore, at the molecular level, the application of sequencing technology to investigate the molecular organization of the brain has become a novel field, and significant progress has been made recently in this field. In this paper, we reviewed the major computational methods used in the analysis of brain transcriptome, including the application of these methods to the research of human and non-human mammal brains. Finally, we discussed the utilization of transcriptome methods in neurological diseases.

## Introduction

Humans and other mammalian species are very different in the aspect of several advanced behaviors, such as language, cognition and sleep. How to explain the differences of these behaviors at the molecular level remains a mystery. The most straightforward idea is that these behavioral differences are the result of many behavior related genes in the human genome, that are not found in other primates, or that the genes responsible for some human specific behaviors and other mammalian animals are quite different in structure. Those differences, at the gene level, lead to different functions responsible for the regulation of behavior. This idea was rejected after obtaining some DNA and protein sequences from humans and humanoid primates such as chimpanzees. For example, by comparing the cytochromes c protein sequence of a human with that of a chimpanzee, most sequences were found to be identical. This finding leads to a conjecture that the difference between humans and other species is not due to differences in their genomic sequences, but mainly because of differences in their regulation and expression (King and Wilson, 1975).

Although there are roughly 20,000 genes in the mouse and human genome (Salzberg, 2018), and about 80% of these genes have significant transcription signatures in the brain (Lein et al., 2007). In the past decade, several important studies have explored the spatio-temporal regulation of gene expression during the brain development of mammals such as mice (Thompson et al., 2014), humans (Colantuoni et al., 2011; Kang et al., 2011; Hawrylycz et al., 2012; Miller et al., 2014) and non-human primates (Bakken et al., 2016; Table 1), using multiple dimensions of brain transcriptomes. Several brain gene expression datasets have been released by different labs or organizations (Lein et al., 2007; Johnson et al., 2009; Kang et al., 2011; Shimogori et al., 2010; Hawrylycz et al., 2012; Bakken et al., 2016). Brain transcriptome atlases have offered great resources to understand the gene expression patterns among different brain regions or during different development stages of a mammalian brain (Mahfouz et al., 2017). With the accumulation of microarray and next-generation sequencing (NGS) data, it is time to explore how the brain is organized at the molecular level. Furthermore, analyses of the transcriptional dynamics of the human brain will afford valuable information to illuminate the molecular activities of gene related brain disorders such as autism (Wu et al., 2016).

**Table 1:** Typical researches on the brain transcriptome of mammals.

Author	Species	Sample size	Neuronal disorders related	Data source link
Lein et al., 2007	Adult mouse	A male, 56-day-old C57BL/6J mice		http://mouse.brain-map.org/
Thompson et al., 2014	Developing mouse	2,100 genes over seven stages of mouse brain development		
Bakken et al., 2016	Developing macaque	(2 males, 2 females) at each of six prenatal developmental stages (E40, E50, E70, E80, E90, and E120)	ASD	http://www.blueprintnhpatlas.org
		Three male specimens at each of four postnatal developmental stages representing the neonate (0 months), infant (3 months), juvenile (12 months) and post-pubertal adult (48 months) were profiled		
Oldham et al., 2006	Human and chimpanzee	Three adult humans and three adult chimpanzees across six matched brain regions		
Konopka et al., 2012	Human, chimpanzee and rhesus macaque	Frontal pole, caudate nucleus and hippocampus of 9 human, 8 chimpanzee and 4 macaque specimens.		
Miller et al., 2014	Prenatal human	Four prenatal human specimens (15pcw, M; 16pcw, F; 21pcw1, F; 21pcw2, F)		http://www.brainspan.org/
Wu et al., 2016	Postnatal human	42 controls and 55 ASD from age 2 to 81.	ASD	
Kang et al., 2011	Developing human	57 human brains spanning from embryonic period to late adulthood		http://hbatlas.org/
Colantuoni et al., 2011	Developing human	269 samples of human prefrontal cortex		
Li et al., 2018	Developing human	1230 samples from 48 brains		http://development.psychencode.org/
Miller et al., 2008	Adult human	31 individuals, comprising nine controls, and 22 AD (data 1) 30 individuals, died of natural causes (data 2)	AD	
Hawrylycz et al., 2012	Adult human	A 24-year-old African American male (Brain 1) A 39-year-old African American male (Brain 2) A 57-year old Caucasian male (Brain 3)		http://human.brain-map.org/
Hawrylycz et al., 2015	Adult human	6 adult humans		http://human.brain-map.org/
Wang et al., 2018	Adult human	1866 individuals	Major psychiatric disorders including ASD, schizophrenia, and bipolar disorder	http://resource.psychencode.org/


Here, we reviewed the computational methods employed to investigate the patterns of gene expression and functional organization of the mammalian brain. We focus our discussion on the analysis of spatio-temporal brain transcriptomes, and we first describe different computational methods such as differential expression (DE) analysis and network analysis. We then describe normal gene networks identified in the brains of mice, non-human primates and humans. Finally, we discuss their potential application to better understand brain diseases. These latest advances have provided a deeper understanding of molecular activities in the brain. Due space constraints, the discussion of this article does not include the single-cell transcriptome, which is a very important emerging field for brain transcriptome analysis. Note that in this review we refer to “transcriptome” as the expression profile of all sets of RNA molecules in one cell or a population of cells and “gene expression analysis” as the investigation of expression profiles using computational approaches.

## Methods of Transcriptome Analysis of the Brain

Most methods of brain transcriptome analysis involve identifying differentially expressed genes, either among normal tissues of various brain regions, or between normal tissues and disease tissues such as autism or schizophrenia. The next step is to study the functions of these differentially expressed genes, as well as network properties, such as their features in the co-expression network. The characterization of these properties lay the foundations to understanding the role of these molecules in the brain.

### Differential Gene Expression Analysis

The aim of differential gene expression analysis is to detect changes of expression levels under different conditions using statistical methods. For microarray data, there are well-established methods such as limma (Ritchie et al., 2015), which uses linear models to detect DE of transcriptomic data, as well as to correct batch effects. For RNA-seq data, two models based on the Poisson distribution and the Negative Binomial distribution are frequently used (Soneson and Delorenzi, 2013). The detailed comparisons of different methods, including the well-designed R package “DESeq” (Anders and Huber, 2010) and “edgeR” (Robinson et al., 2010), was discussed in a previous review (Soneson and Delorenzi, 2013).

The rapid development of high-throughput techniques, such as microarrays and NGS, makes it possible to assess the status of a cell’s transcriptome at any given time (Barabási and Oltvai, 2004). Several methods are applied to analyze the transcriptome data. Traditional methods involve comparisons of knockout with wildtype samples, or of diseases with control groups. Several pilot studies have provided a first glimpse of the brain transcriptome, mainly with the DE gene methods, to compare knockout with wildtype mice (Geschwind and Konopka, 2009). In the first step, an analysis of DE is performed, and DE genes are identified. Next, functional annotation of these DE genes can be assessed by gene ontology (GO) enrichment or KEGG pathway analysis, and enrichment of disease candidate genes can also be performed. However, as the brain is a complex network system composing of multiple cell types, it is claimed that DE analysis may not be sufficient to obtain the underlying structure of gene expression data from the central nervous system (Miller et al., 2008; Oldham et al., 2008; Winden et al., 2009; Konopka, 2011).

### Network Analysis

Network-based methods are proven to be more powerful than absolute magnitudes of expression levels, in revealing gene expression patterns (Oldham et al., 2006; Miller et al., 2014; Hawrylycz et al., 2015, 2012), and have been found useful in analyzing the inner workings of a cell (Wang and Huang, 2014). Using network analysis, we can study higher order properties of brain transcriptome.

Gene expression profiling data can be modeled as a network, in which each gene corresponds to a node and gene pairs are connected by an edge if their expression values are highly correlated (Parikshak et al., 2015). In a network, degree is an elementary characteristic of a node, and the degree distribution indicates the probability that a selected node has exactly N links. The nodes’ degrees in random networks follow a Poisson distribution, while most biological networks approximate a scale-free topology, which means that fewer nodes are highly connected and most nodes have low connectivity. Biological networks exhibit a high clustering feature and consist of a set of modules, where several nodes form a densely connected community have sparser connections with the rest of the network. Within functional modules, cellular functions are executed by clustered molecules (Barabási and Oltvai, 2004).

**FIGURE 1:**
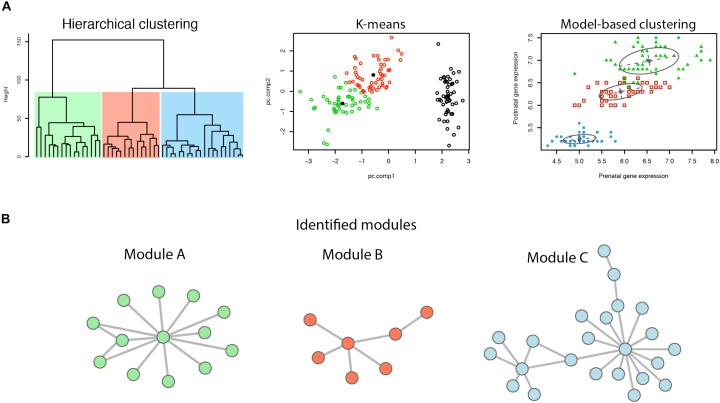
Identification of network modules. **(A)** Illustration of different clustering methods. **(B)** Modules identified by clustering of genes.

Co-expression of genes is defined as genes with similar expression patterns. Common measures of gene co-expression include Pearson correlation, Spearman correlation, Euclidean distance, and the angle between a pair of observed vectors (D’haeseleer et al., 2000; Horvath and Dong, 2008). In a gene co-expression network, modules refer to sets of highly co-regulated genes (Barabási and Oltvai, 2004). To identify gene modules, several clustering methods have been employed, including hierarchical clustering, model-based clustering, k-means, etc. (Figure 1). Genes within a module work together to achieve a distinct function.

One major goal of co-expression network analysis is to identify gene modules (Barabási and Oltvai, 2004). Gene co-expression patterns of the brain are mainly evaluated by correlation-based measurements (Mahfouz et al., 2017). By detecting similar gene expression patterns to disease genes, *in silico* prediction can be made with the gene co-expression approach. To discover clusters of co-expressed genes within a set of samples, a commonly used unsupervised method is hierarchical clustering (Mahfouz et al., 2017). One method used to identify co-expression modules is Pearson correlation, the most popular co-expression measure (Wang and Huang, 2014), as the distance measurement for hierarchical clustering. Hard thresholding is then applied to produce a network (Li et al., 2016).

One widely used method for co-expression network construction is weighted correlation network analysis (WGCNA), which was first introduced by Zhang and Horvath (2005). It is an informative method for detecting biologically relevant patterns using high-dimensional data sets, and it allows for the assessment of the relation of modules to experimental traits (Zhang and Horvath, 2005). Genes with strongly covarying patterns are grouped into modules across the sample set. Identified modules are characterized by module eigengenes, and hub genes refer to genes that are highly correlated with the eigengenes. WGCNA is a systems biology method used to construct modules of gene co-expression with an unsupervised clustering approach and has been broadly applied to transcriptome analysis of the mammalian brain (Oldham et al., 2006; Hawrylycz et al., 2012; Thompson et al., 2014; Bakken et al., 2016). WGCNA searches for gene modules of co-expression with high topological overlap (Zhang and Horvath, 2005). First, a soft thresholding power is chosen to calculate adjacency, which is further transformed into a topological overlap matrix. Then, the dendrogram of genes can be produced through hierarchical clustering. Finally, modules are identified using a dynamic tree cut method for branch cutting (Langfelder and Horvath, 2008).

Besides the popular WGCNA, there are also a number of different methods that have been developed for cluster analysis and further detecting network modularity analysis (Figure 1). The K-mean clustering method sets the number of clusters (K) before clustering, and then, based on the calculation of distance (typically Euclidean distance), all different modules are detected (Jain, 2010). However, different cluster initialization may lead to different final clustering. Another plausible approach is based on probability models, the network nodes of which are calculated based on the probability distribution of the genes (Yeung et al., 2001). The model-based method can capture correlation and dependence between attributes, and is implemented in the R package “mclust” (Yeung et al., 2001).

## Analysis of Normal Mammalian Brains

### Mouse Brain

For decades, mice have been used as a model organism to study human biology and diseases (Breschi et al., 2017). It is claimed that transcriptional patterns between orthologous organs of different species are more similar than those between different organs from the same species (Brawand et al., 2011; Barbosa-Morais et al., 2012; Merkin et al., 2012; Breschi et al., 2017). Therefore, mouse brain transcriptome data are very useful to complement the study of the human brain and neuronal disorders, as a series of processes of primate brain development are conserved across mammals (Bakken et al., 2016).

Using voxel expression data, Thompson et al. (2014) explored the temporal co-expression patterns of the mouse brain in the diencephalon over three time periods: “embryonic,” “postnatal” and “all.” They analyzed the “all” period and found that genes in two modules showed strong upregulation in the diencephalon at P14 and P28. They further examined the postnatal cluster and found that a set of well-known oligodendrocyte genes were not widely distributed until P14. An especially interesting temporal expression pattern was that P14 exhibited strong thalamus-specific expression of predominantly TF genes. The authors inferred that this may coincide with eye opening and the initial reception of visual stimulation by the thalamus.

### Non-human Primate Brain

Despite the fact that humans and mice share many core biological processes and genetic elements, many human brain features are poorly modeled in rodents (Bakken et al., 2016) due to the extended periods of primate brain development. Compared with rodents, humans and monkeys are more similar on expression trajectories of brain development (Bakken et al., 2016). In addition, the comparison of co-expression patterns between human and chimpanzee brains, showed that many hub genes in the human brain are conserved in the chimpanzee brain (Oldham et al., 2006).

Bakken et al. (2015) explored the spatio and temporal expression patterns of a postnatal brain of a rhesus monkey. Five brain regions were considered for the genome-wide gene expression at birth, infancy, childhood and young adulthood. They identified 27 modules in total. Correlating each module eigengene with age and brain region, they found several age-related modules, with a gradual shift of gene expression postnatally. They also identified cortical area-specific expression modules such as the primary visual cortex enriched module (M6). They explored the expression of M6 genes, and confirmed the previous finding that, in rhesus monkey and adult human brains, the gene expression pattern in the primary visual cortex is distinct from that of other brain regions.

However, in the cerebral cortex, there are prominent differences between humans and chimpanzees, consistent with the expansion of the cortex in the human lineage (Oldham et al., 2006). Moreover, Zhu et al. (2018) compared the development of the nervous system between humans and macaques, and detected a cup-shaped pattern of transcriptomic differences between the two species. In addition, they also identified human-distinct gene co-expression modules, indicating the difference of molecular mechanisms for species divergence, which could play a role in mental disorders. Therefore, to reveal human-specific features of the brain at the molecular level, it is necessary to use human brain transcriptome instead of a non-human primate brain transcriptome.

### Human Brain

Human brain development is a complex process and depends on the precise regulation of gene expression (Rakic, 2009; Rubenstein, 2011). Using transcriptome data of highly differential stability genes, Hawrylycz et al. (2015) constructed a consensus gene co-expression network and found several modules with the most neuronal function-related annotations. Allocating genes to each of the identified modules according to the gene’s correlation to the corresponding module eigengene, they detected a number of modules which were remarkably selective for certain brain regions. Interestingly, when assessing the module preservation between humans and mice, they found that some neuron-related modules were well preserved, whereas many of the most non-neuronal modules were poorly preserved. Nevertheless, several genes differ in their expression patterning across species even in highly preserved modules. Modules associated with neurons were better conserved than modules associated with glia.

Using data from 16 regions comprised of six brain structures across pre- and postnatal development periods, Kang et al. (2011) created a gene co-expression network and identified 29 modules related to different spatio-temporal profiles. They found that 90% of the expressed genes were differentially regulated at the whole-transcript or exon level across brain regions or brain development periods. Among these modules, M8 showed the highest expression levels in the early fetal neocortex and hippocampus, and then a progressive drop in expression levels until infancy. The hub genes of M8 are involved in the development of the neocortex and the hippocampus projection neurons. In addition, they identified two temporally regulated modules, with opposite developmental trajectories: M20 showed decreased expression while M2 showed increased expression, with the shift just before birth, which indicates that environmental influences are probably associated with the transcriptional changes at this period of brain development.

Furthermore, using gene expression data of 11 neocortex areas in human and macaque brains, Pletikos et al. (2014) analyzed the spatial expression patterns among areas across development periods. They first applied the ANOVA approach to identify differentially expressed genes among neocortex regions, at each development period and proposed an hourglass model of interareal transcriptional divergence over time, indicating that the spatial pattern of interareal divergence is primarily driven by a number of functional areas. In addition, to gain insight into the organization of the neocortex transcriptomes, they further performed WGCNA with samples from two periods (fetal development period and from adolescent period onward) of increased interareal differences and identified 122 modules and 207 modules, respectively. Most of the fetal modules showed temporally specified areal patterns and lost their prominent areal differences postnatally. In contrast, adolescent and adult modules were more stable over time, and showed less complex spatial patterns.

Moreover, Li et al. (2018) integrated transcriptome, DNA methylation, and histone modifications data from 16 brain regions, and revealed a cup-shaped pattern of regional divergence during prenatal and postnatal development. Specifically, they identified a group of gene co-expression modules associated with dynamic spatiotemporal trajectories and uncovered that many modules are enriched with specific cell types or disease-associated genes.

Using organoids from human pluripotent cells, Amiri et al. (2018) modeled the cerebral cortical development between 5 and 16 weeks post-conception. They identified the networks of genes and enhancer modules and found that some enhancer modules converged with gene modules, indicating the regulation of co-expressed genes by enhancers across time.

## Mental Disorders

Integrating co-expression network analysis to traditional differential gene expression analysis uncovered features of normal mammalian brains and expanded our knowledge of the spatio-temporal event in mammalian brain development over the last decade. Moreover, in order to reveal the molecular mechanisms of neuronal disorders, such as Autism spectrum disorder (ASD), Alzheimer’s disease (AD), Schizophrenia, etc., co-expression networks are applied to compare healthy and diseased brains, which would also reveal important biological pathways in these disorders and provide potential biomarkers or therapeutic targets (Keo et al., 2017; Seyfried et al., 2017; Mostafavi et al., 2018; Rajarajan et al., 2018).

Utilizing gene expression analysis to decode the mechanism of mental disorders is a powerful tool as it is large-scale, high-throughput and cost-efficient. ASD is a group of neurodevelopmental disorders characterized by deficits in social functioning and repetitive, restricted behaviors or interests (Bourgeron, 2015). Previous findings show that ASD genes are enriched only in pathways during early fetal development (Parikshak et al., 2013). In the networks of a postnatal rhesus brain, Bakken et al. (2015) found that ASD gene enriched modules show significant enrichment in the neocortex. Gene expression in one of these modules was high in the neonatal cortex and striatum but low during infant and juvenile development periods. Combining dense temporal sampling of prenatal and postnatal periods, Bakken et al. (2016) demonstrated a high-resolution transcriptional atlas of macaque (*Macaca mulatta*) brain development with fine anatomical division of cortical and subcortical regions associated with human neuronal disease. They found that many ASD genes exhibited a coordinated expression in postmitotic neurons both prenatally and postnatally. They also found that in neuronal progenitor-enriched modules, MCPH genes were enriched in early- to mid-fetal ages. No enrichment of intellectual-disability-associated genes was observed in any modules. Using 109 cortex miRNA samples, Wu et al. (2016) applied WGCNA and identified 11 modules. By examining the relationship between module eigengene and ASD traits, they detected three modules significantly correlated with ASD, and successfully predicted and validated two transcription factors which regulate neuronal genes in ASD.

Alzheimer’s disease is the most common cause of neurodegenerative dementia (Verheijen and Sleegers, 2018). Using 19 cortical regions, Wang et al. (2016) constructed region-specific co-expression networks, and rank-ordered co-expression modules and brain regions based on their association with AD pathological traits. They found that temporal lobe gyri exhibited the largest and earliest gene expression abnormalities. Mostafavi et al. (2018) applied a network-based method and identified specific genes that were associated with AD-related traits. By integrating clinical, neuropathology and gene expression data, they detected a co-expression module which is related to both cognitive decline and β-amyloid burden. Furthermore, they identified two genes in the module, *INPPL1* and *PLXNB1*, as potential AD therapeutic targets.

Gandal et al. (2018a) analyzed the transcriptome of five major psychiatric disorders, including ASD and schizophrenia, and identified a number of shared and disorder-specific co-expression modules. They found an up-regulated module, which is associated with astrocyte, and several down-regulated modules, which are annotated as neuronal or mitochondrial, across ASD, schizophrenia, and bipolar disorder, suggesting pathways of molecular convergence of major neuropsychiatric illness.

Nevertheless, the PsychENCODE consortium integrated multiomics data and provides a comprehensive resource for the functional genomics of the human brain (Wang et al., 2018). For example, Gandal et al. (2018b) integrated RNA-seq and genotypes in brain samples with ASD, schizophrenia, and bipolar disorder, and detected gene co-expression modules related to each disorder. They found that one module, associated with the microglial cell marker, is up-regulated in ASD, and down-regulated in schizophrenia and bipolar disorder, suggesting a previously unrevealed neural-immune mechanism.

Integration of co-expression data with clinical traits enables the identification of novel disease related modules and hub genes, which provide potential therapeutic targets for related neuronal disorders.

## Conclusion and Further Direction

Transcriptomic data of the mammalian brain provides eminent opportunities to illuminate how the brain works in the molecular level. The current status of this field has provided us with great insight on the molecular developmental patterns of the brain, and we expect more primate brains to be included in future research. Additionally, other molecular activities such as microRNA and non-coding RNAs should be profiled at the brain-wide scale as well. In this article, we summarized the progress made by various researchers in the analysis of brain transcriptome in recent years. In addition to traditional DE analysis, network-based methods offer an unsupervised perspective to analyze large scale data from mouse to human brains, as well as data of different developmental stages of each species. Moreover, systems-level analysis assembles correlates single genes and enables the discovery of key pathways. As the rapid development of NGS in the past decade has accelerated the research on transcriptomics of the brain, the knowledge obtained from this field can facilitate deciphering the complexity of the brain and help us gain valuable insight into the organization of the brain’s functions. Nevertheless, the use of network-based methods integrated with clinical traits and experimental validation (Mostafavi et al., 2018) demonstrates a blueprint for investigating complex neuronal diseases.

One major limitation of bulk sample transcriptome analysis is that it can’t provide insight into the behavior of different cell types, which is a critical aspect of brain research. Similarly, the analysis methodologies developed for bulk samples may not be suitable for analyzing single-cell data with algorithms of a network. In this mini-review, the recent emerging single-cell sequencing data is not covered due to the space constraints. The analysis of differentially expressed genes between different cell types or of the marker between different cell types would be an important topic in the future. Research in this area is progressing rapidly (Zeisel et al., 2015; Tasic et al., 2016, 2018; Fan et al., 2018; Kelley et al., 2018; Zhong et al., 2018), and we look forward to some critical improvements for the identification of cell types related to differentially expressed genes in the future.

## Author Contributions

WW drafted the manuscript. WW and G-ZW finalized the manuscript.

## Conflict of Interest Statement

The authors declare that the research was conducted in the absence of any commercial or financial relationships that could be construed as a potential conflict of interest.
